# Predicting the Optimal Input Parameters for the Desired Print Quality Using Machine Learning

**DOI:** 10.3390/mi13122231

**Published:** 2022-12-16

**Authors:** Rajalakshmi Ratnavel, Shreya Viswanath, Jeyanthi Subramanian, Vinoth Kumar Selvaraj, Valarmathi Prahasam, Sanjay Siddharth

**Affiliations:** 1 School of Computer Science Engineering, Vellore Institute of Technology, Chennai 600127, India; 2 School of Mechanical Engineering, Vellore Institute of Technology, Chennai 600127, India

**Keywords:** machine learning, additive manufacturing, fused filament fabrication, design of experiments, Taguchi

## Abstract

3D printing is a growing technology being incorporated into almost every industry. Although it has obvious advantages, such as precision and less fabrication time, it has many shortcomings. Although several attempts were made to monitor the errors, many have not been able to thoroughly address them, like stringing, over-extrusion, layer shifting, and overheating. This paper proposes a study using machine learning to identify the optimal process parameters such as infill structure and density, material (ABS, PLA, Nylon, PVA, and PETG), wall and layer thickness, count, and temperature. The result thus obtained was used to train a machine learning algorithm. Four different network architectures (CNN, Resnet152, MobileNet, and Inception V3) were used to build the algorithm. The algorithm was able to predict the parameters for a given requirement. It was also able to detect any errors. The algorithm was trained to pause the print immediately in case of a mistake. Upon comparison, it was found that the algorithm built with Inception V3 achieved the best accuracy of 97%. The applications include saving the material from being wasted due to print time errors in the manufacturing industry.

## 1. Introduction

Additive manufacturing (AM), or 3D printing, is one of the most promising manufacturing techniques. Several industries have started to incorporate this technology. Quickly producing geometrically intricate and complex designs is one of the critical advantages of this technology. Different categories of AM are differentiated based on the composition of the raw material being used. Some examples of liquid-based AM are fused filament fabrication (FFF), polyjet, and stereo lithography (SLA). At the same time, laminated object manufacturing (LOM) is a solid-based AM. Examples of powder-based AM include selective laser sintering (SLS), electron-beam additive manufacturing (EBM), and LENS [1].

Among all the AM techniques, FFF is the most commonly used one. Its ability to fabricate geometrically complex parts with precision makes it very versatile. Initially developed by Stratasys, FFF works on the principle of layer-by-layer addition of molten thermoplastics. The thermoplastic filament is fed into the liquefier using a filament-pulling system, which uses rollers to wind the filament down. Then, a liquefier was used to soften and melt a filament by heating it above its melting point. The molten filament is then pushed through a nozzle. The extruded polymer is deposited onto the bed as the liquefier moves [2,3,4,5]. The bed is relatively cooler, allowing the molten plastic to stick onto the bed and cool down enough to bond with the subsequent layer, forming a rigid product. Several parameters affect the quality of the print. These factors include material, nozzle and bed temperature, filament thickness, nozzle diameter, infill structure and density, retraction, layer thickness and count, and wall count and thickness. The 3D printing process can be conducted in hot and cold rooms as long as the printer’s temperature is controlled and appropriate material is used. While most printing occurs efficiently in hot environments, there are a few limitations. 

For example, according to [6], materials like polylactic acid (PLA) would not do well in a hot atmosphere(temperatures exceeding 35 °C), as they would not have enough time to harden. On the other hand, materials like ABS are not sensitive to temperature changes and are easy to work with in a hot atmosphere due to thermal and chemical properties; PETG combines the strength of ABS with the simplicity of PLA; Nylon has a low coefficient of friction with a high melting point, making it useful for support structure material. These materials are cheap and offer relatively competitive mechanical properties compared to conventional manufacturing materials. According to the authors of [6], regardless of the environment, it is essential that the temperature of the printing atmosphere is controlled and maintained at an appropriate temperature depending on the material being printed. Extreme hot conditions might deform the structure, and coldness may lead to warping. Hence, we can conclude that room temperature significantly influences the quality of the printed product. The temperature of the extruder and heating bed also plays an essential role in the print quality. It is necessary to set the temperature to an optimal value depending on the material used. According to the authors of [7], one can set the temperature of the heated bed and select the extruder’s temperature based on the material used. Wall thickness and count, layer thickness and count also significantly affect mechanical properties. When the thickness and count increase, the strength and stiffness of the product also increases. However, the weight of the fragment also increases. This may not be favourable, as automotive industries want lightweight products with enhanced mechanical properties.

Apart from the material’s melting point, other properties such as mechanical, and physical parameters should be considered when choosing a material [8]. Mechanical properties such as tensile and flexural strength, impact loading, modulus of elasticity, and rigidity are crucial. For instance, the authors of [9] found that a part fabricated with ABS material, kept at 0 raster angle, showed the best tensile and flexural strength. Similarly, Garg et al. [10] found that tensile strength highly depended on the building orientation. They found that a part built flat on a long edge showed higher tensile and flexural strengths than a piece made flat on a shorter edge. This validates the importance of process parameters in determining the quality of the printed product. For instance, Qattawi et al. [11] studied other parameters such as infill structure and density, print speed, and layer height. Their study focused on how the parameters mentioned above affected the mechanical properties of the fabricated part. The common consensus among the scientific community is that cubic, cubic subdivision, and honeycomb structures are the best infill structures to achieve high stiffness and strength. One can use machines like the universal testing machine (UTM) to perform tensile and compressive tests. One can perform Izod and Charpy tests to determine the impact strength. Different apparatuses are required to perform various tests; this can be tedious and time-consuming. Finite element methods help overcome that, provided you have a system with strong computational power. One can use software like ANSYS to simulate and perform the abovementioned tests. The analysis offers an accurate solution to the entire model by undertaking an element-wise approach [12,13,14,15]. Abbot, D. W. et al. [16] used finite element methods to study the strength and toughness of a rectangular block using compression tests. They varied infill structures and materials to get different samples. Given the number of factors and the values it can take, making test specimens with different combinations of parameters can be difficult. The Design of Experiments (DOE) can help plan, design, and analyze experiments [17,18]. It is a combination of statistical and mathematical functions where studies of how a response is affected by one or more factors are conducted. DOE has been traditionally used to improve reliability and quality [18]. Some examples of DOE are Taguchi, Response Surface Methodology (RSM), screening, and factorial and mixture designs. RSM and Taguchi have not only been incorporated in the manufacturing and engineering industries [19,20,21] but also in pharmaceuticals [22], food [23], hospitals, architecture [24], and energy industries. It can also be incorporated into computer simulation models [25]. 

Today, in the wake of the pandemic, contactless manufacturing embedded with machine vision and artificial intelligence is vital. A field of computing algorithms called machine learning is constantly developing and aims to replicate human intelligence by learning from the environment [26]. Several network architectures are available. For instance, the CNN (convolutional neural network) algorithm is the most well-known and often-utilized. CNN’s main benefit over its predecessors is that it automatically recognizes the essential components without human intervention. Similarly, a deep convolutional architecture called the Inception v3 Network was created for classification tasks using the ImageNet dataset, which consists of 1.2 million RGB pictures from 1000 classes [27]. Another network architecture, the MobileNet Application Programming Interface (API), was created to improve accuracy while using little power and responding quickly. MobileNet can quickly address various problems related to instance segmentation, object recognition, and image/object classification. Several researchers have devised ways to detect, monitor, and correct these defects using machine vision and machine learning. Concepts of machine learning, when coupled with the internet of things, help promote contactless manufacturing. Appropriate sensors can be employed to monitor and correct the print in the event of an error. For instance, the authors of [12] created a CNN-based model to detect mistakes in 3D printing. They achieved reasonably good accuracy and efficiency. The lead researchers of [13] used machine vision and statistical process control (SPC) to monitor the print. They reached an accuracy of 0.5mm. Similarly, the authors of [14] used support vector machines to process the data collected from accelerometers, thermocouples, and acoustic emission sensors to monitor the fabrication process [28,29]. 

This paper presents a novel machine-learning algorithm that predicts the optimal input parameter based on the users’ wishes and monitors and controls the print. The algorithm was trained using four network architectures: CNN, MobileNet, Resnet152, and Inception_V3. RSM, and Taguchi’s method was used to determine and design the experiment. Print parameters such as infill type, density, material, wall and layer thickness, and count were varied to design the experiment. Finite element analysis is performed using Ansys 2022 software (Ansys Inc., ARK Infosolutions, Pvt.Ltd., Chennai, India) to understand the mechanical properties (strength, and stiffness) of all the test specimens. The inferences obtained from the finite element analysis are used to train the novel machine learning model. This machine learning model will help save time, as companies and manufacturers will not have to spend time and resources to find the optimal input parameters to obtain the desired outputs. This can be implemented in almost every manufacturing industry.

## 2. Neural Networks

### 2.1. Convolutional Neural Network (CNN)

CNN is the most well-known and often-utilized algorithm [30]. CNN has a significant advantage over its predecessors because it automatically recognizes relevant elements without human intervention [31]. CNNs have been widely employed in several domains, including computer vision, audio processing [32], facial recognition, etc. In most situations, a photograph is utilized as the input for CNN-based object classification algorithms, producing the class type [33,34]. Using an R-FCN(Region-based Fully Convolutional Networks) network, an auto-sorting system with a 97% accuracy rate that could be employed on an assembly line conveying diverse parts was developed [35]. It can extract a sequence of convolutional feature maps from an image of a component. These feature maps reflect essential visual characteristics and are utilized for object classification and localization. The network provides a collection of bounding boxes {{P An = P Ax n, P Ay n, P Aw n, P Ah n}}, each of which contains one item recognized. Each bounding box specifies an element picture for the following phase.

In the first convolutional layer of CNN, the weights of the receptive field and the feature map may be utilized to determine the variable and temporal information required for fault classification. During CNN’s training phase, weights were modified using the gradient descent technique to lower the loss (classification error) function value. This implies that when the size of a single weight increases, the last nodes contribute more significantly to extracting information relevant for classification. Each weight column of the CNN model’s first convolutional layer reflects the importance of a particular sensor variable [36].

### 2.2. MobileNet

MobileNet, created by Google, is a solid deep neural network that seeks to perform well. The Application Programming Interface (API) was designed to improve accuracy while consuming little power and responding quickly. MobileNet can help with instance segmentation, object identification, and image or object classification challenges, to name a few [37]. MobileNet is available in four variants: mobilenetv1, mobilenetv2, mobilenetv3, and mobilenetedgeTpu. The variants are evolutions of the previous models that aim at making the training process less complicated by using better separable convolutions. Mobilenetv2 is utilized in this paper.

In this system, depth-wise separable convolutions, the first layer of the architecture, are used to filter light. The next layer is a point wise convolutional layer with a dimension of 1*1. The latter is in charge of developing new characteristics by employing certain linear combinations on input layers. Relu6, which provides computation robustness but low precision, is used [38]. Spontaneous layer segregation benefits bottleneck and transformation layers. The inverted residual layer provides a memory-efficient implementation. In this article, the MobilnetV2 model is integrated with the Input Layer, Functional MobileNet Layer, and Global Average Pooling Layer to form a dense layer.

### 2.3. InceptionV3

The InceptionV3 is a convolutional neural network for helping in image analysis and object detection. The InceptionV3 was designed to approach deep and broad networks by reducing the number of parameters [39]. The InceptionV2 experimenters observed that auxiliary classifiers did not contribute much until the end of the training process. Thus, the V2 was improved without drastically changing modules. It was achieved by adding an RMSProp optimizer, factorized 7 × 7 convolutions, batch norm in auxiliary classifiers, and label smoothing. The RMSProp optimizer limits the oscillations in a vertical direction, thereby increasing the learning rate, and the algorithm may converge faster as it takes longer steps in the horizontal direction.

Factorization of convolutions and reducing high dimensions inside the network can result in relatively low computational effort and better performance. Batch normalization is a method to standardize the inputs to each layer of the mini-batch, thus neutralizing the learning process and reducing the number of training epochs to train deep networks [34,40].The present-day application of InceptionV3 has increased enormously across various domains, from healthcare [41] to robotics, and archaeology [42].

### 2.4. Resnet152

A Residual Network(Resnet) is a CNN architecture. It has blocks of CNN in multiple layers. It is one of the most popular and successful deep learning models. Residual networks help ease the training of deeper networks [43]. This makes it possible to train hundreds or even thousands of layers and achieve compelling performance. Increasing the depth does not provide us with better performance. Deep networks are hard to train due to the vanishing gradient problem. The version becomes saturated or degraded when the grid becomes more profound. Resnet provides a way to handle the vanishing gradient problem by skipping one or more layers (identity shortcut connection) so that not all coatings are executed. It helps eliminate the vanishing gradient problem. The authors of [43] argue that combining layers should not degrade the network performance.

A deeper model can still achieve the required performance by skipping layers despite the vanishing gradient problem. According to the authors of [38], removing a few layers in a Resnet architecture does not compromise its performance.

## 3. Methodology

### 3.1. Fused Filament Fabrication (FFF)

**Figure 1:**
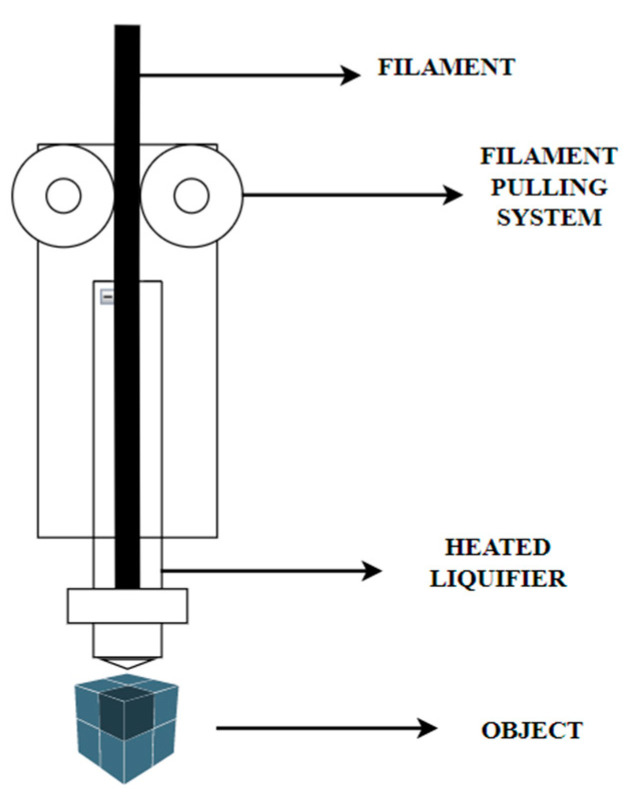
FFF process visual representation.

The thermoplastic filament is fed into the liquefier using a filament pushing system, which then uses a set of rollers to the filament down. Then, a liquefier is used to soften and melt a filament by heating it above its melting point. Table 1 represents the thermoplastics melting point range. The molten filament is then pushed through a nozzle. The extruded polymer falls onto the bed as the liquefier moves. The bed is relatively cooler, allowing the molten plastic to stick onto the bed and cool down enough to bond with the subsequent layer, forming a rigid product. The visual representation of FFF process is shown in Figure 1.

### 3.2. Design of Experiments—Taguchi 

Five levels are considered for each of the process parameters. The Taguchi method is used to develop and design an experimental model with different permutations and combinations of infill structure and density, material, wall and layer thickness, and nozzle diameter. The Taguchi method is efficient as it gives the correlation between the variables and the response with a minimum error. A total of 25 test specimen combinations are represented. Each level is labelled as 1, 2, 3, 4, and 5. Table 2 describes the parameter levels used for the DOE, whereas Table 3 specifies the different combinations of the input parameters. 

### 3.3. Modelling and Finite Element Analysis

**Figure 2:**
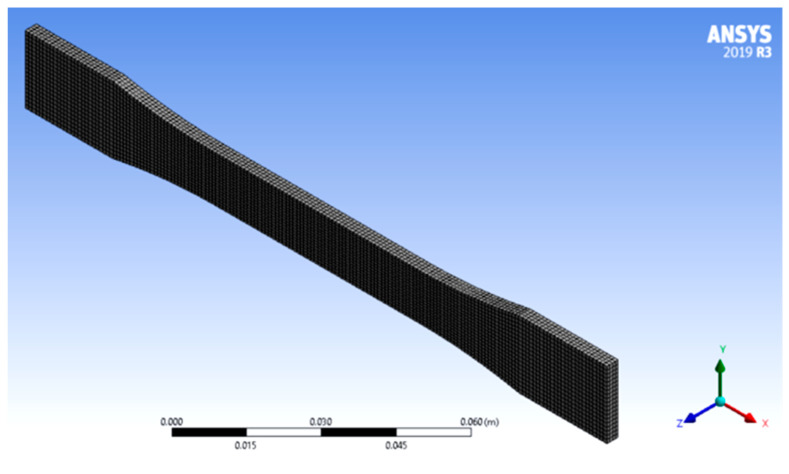
Modelling and meshing of sample using Solidworks.

Solidworks 2022 software, (Dassault Systemes Corp., Sim Technologies, Chennai, India) was used to design and model the test specimen (Figure 2). A standard ASTM model (ASTM D638) was created using the above software. The model wasthen sliced according to the specifications in Table 3 using Ultimaker Cura (5.0, Ultimaker, India). The software stores the models in the “.stl” format. Finite element analysis includes tensile, compressive, and fatigue tests. The model meshed with an element size of 1 × 10^−4^ m.The meshed model had 23.7 × 10^6^ meshes. For the tensile test, one of the end plates of the test specimen was fixed, and a load of 1000 N was applied on the opposite end and direction. Similarly, a load of 1000 N was applied on both ends in the same direction for the fatigue test. The fatigue test helps determine the creep, which helps find the specimen’s life. The tensile and compressive tests help find the material’s breaking point. The results thus obtained are used to train the machine learning algorithm.

### 3.4. System Design 

**Figure 3:**
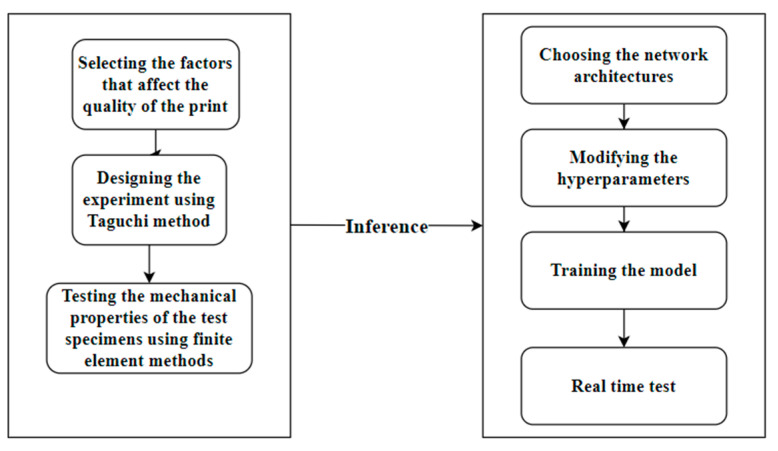
Methodology for training the machine learning algorithm.

The machine learning algorithm was trained using the inferences obtained from the finite element analysis. It is essential to design the experimental setup before training the algorithm. Sensors such as accelerometers, thermocouples, and infrared cameras are connected to the 3D printer. The algorithm is connected to the printer using Raspberry Pi 4 and Arduino Uno (R3, Arduino, Italy, Chennai, India) The model and the sensors(accelerometers with range: −g to +g, thermocouples with range 32 to 5300° F, and infrared cameras) are connected. The algorithm is prepared to send appropriate input parameters to the 3D printer (Sedaxis Advanced Materials Pvt. Ltd., Chennai, India) based on the user’s requirements and the inference from the finite element analysis. For instance, if the user wants a lightweight and flexible product, then the algorithm is trained to choose Gyroid as the infill pattern and PLA as the material. The algorithm also gets feedback from the system to monitor the print. In the event of an anomaly, the algorithm takes appropriate measures to ensure the smooth fabrication of the process. Figure 3 shows the methodology for training the machine learning algorithm.

### 3.5. Training the Machine Learning Algorithm 

**Figure 4:**
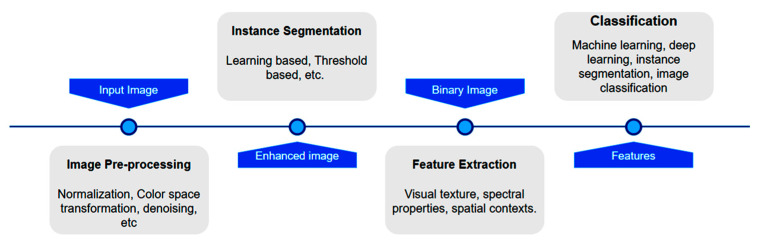
General workflow of the image pre-processing process for image classification.

**Figure 5:**
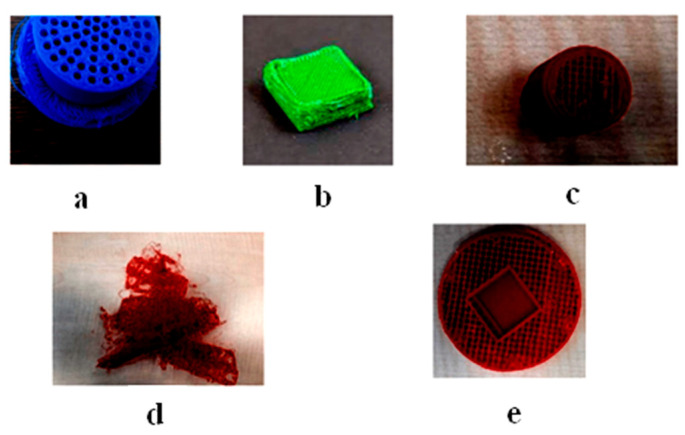
Common errors in 3D printing (**a**) Stringing (**b**) Over-extrusion (**c**) Under-extrusion (**d**) Pillowing (**e**) Layer Shifting.

First, the data generated from the finite element studies were enhanced. The custom data set consists of images of simulated errors and limited element studies. Data must undergo checking before being trained. Blurred images, unwanted noise, and merging contours are a nuisance. Thus, the images must be pre-processed and enhanced to make the dataset as valuable as possible. Generally, normalizing, denoising, resizing, contrast enhancement, and colour space transformation fall under the purview of image pre-processing [25]. The general workflow of the image pre-processing process for image classification is shown in Figure 4. It is of utmost importance to have all images in the dataset be the same size. Thus, resizing images is a necessary task. It also helps to train at a faster rate. Here, the inferences obtained from the finite element analysis and the final print images with and without simulated errors (Figure 5) were used as the input to train the machine learning algorithm. Next, one must tune the hyper parameters to the optimal setting to achieve the minimum loss function and accuracy. A feature extractor is responsible for feeding the input for the training process. It picks notable features (Regions of Interest, or ROIs) from the given data and feeds them into the model for training. “Train_ROIs_Per_Image” refers to the number of regions of interest that can be isolated and trained per image. The model allows 100 ROIs per image. The loss function is the summation of errors after every training step. A loss can be classified as a class loss, bounding box loss (bbox_loss), or region loss (Region_class_loss). The summation of all these three loss functions gives the overall loss function. Initially, the loss function was set as 1, the highest value a loss function can take. After proper training, the preferred loss function value was less than 0.05.

Once all the preliminary checks are done, the model must be trained. Every neuron in a neural network is associated with hyperparameters such as weight (w) and bias (b). The consequences must be pre-trained on other datasets for a transfer learning approach. The aim of training the custom model from scratch is to find the optimum value for weights and biases for each neuron in the network. The former and the latter can be fine-tuned to attain maximum precision and accuracy. The temporarily assigned hyper parameter values are updated with new values that minimize the error function during back propagation. A hundred epochs are performed to obtain the ideal hyperparameters that minimize the loss function. The desired loss function value is less than 0.05. Once the model undergoes sufficient training to achieve the value for the loss function, a script was used to convert the weights into a Tensor Flow frozen inference graph. Once converted, the inference was run on some test images, and masks were generated at a high level of accuracy.

### 3.6. Hyperparameters of Chosen Network Architectures

This paper uses four different network architectures to train the novel machine learning algorithm. The four models are then compared based on their precision and accuracy. Table 4 mentions the hyper parameters of the chosen architectures with results. The hyper parameters of all four network architectures are mentioned below:

- Learning Rate: The amount by which weights are updated after each epoch is referred to as the learning rate. This value typically ranges between 0.0 and 1.0. The lower the learning rate, the higher the training time, and vice versa. However, extremely high values are not preferable as the coverage might be constrained, so the resulting accuracy may not be optimal.
- Optimizer: Optimizers help update the weights along with other parameters. The model’s performance is highly dependent on Optimizer.
- Batch Size: The interval of examples after which model parameters must be updated. This value must be greater than or equal to 1 but less than the number of training examples.
- Activation function: These functions determine the neural network’s output and map it into its range.
- Epochs: This refers to the number of times a model learns and updates itself during training.
- F1 Score: This is a means to evaluate and express the model’s performance and classifier.

## 4. Results and Discussion

### 4.1. Regression Equation and Optimization 

The obtained regression equation is a first-order polynomial equation. Corresponding material values, will thickness, infill pattern, infill density, nozzle diameter, and layer thickness are substituted in the regression equation.

Deformation = 0.00364 + 0.001706 A+ 0.000260 B − 0.000025 C − 0.00860 D − 0.00171 E+ 0.00262 F (1)

Factor of safety = 6.48 − 0.782 A + 0.044 B + 0.0246 C+ 4.04 D+ 0.10 E − 1.60 F (2)

Equivalent Stress = 18.3 − 1.86 A − 0.08 B + 0.056 C+ 15.0 D + 1.2 E − 2.6 F(3)

where A = Material, B = Infill Structure, C = Infill density, D = Wall thickness, E = Layer thickness, F = Nozzle diameter. 

**Figure 6:**
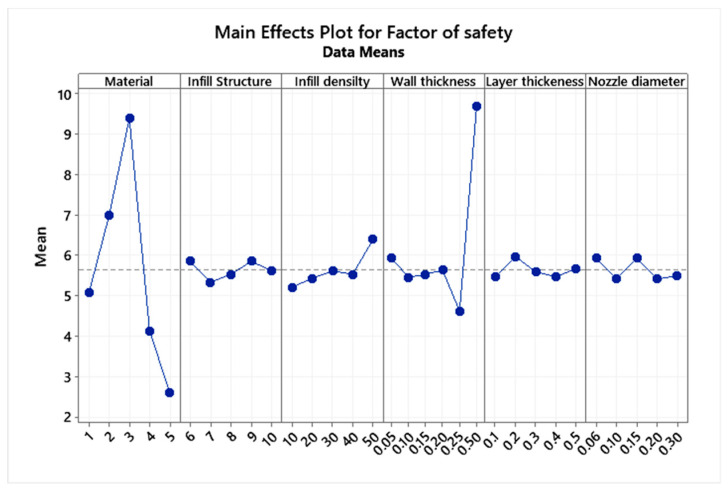
Main effects plot for the factor of safety.

**Figure 7:**
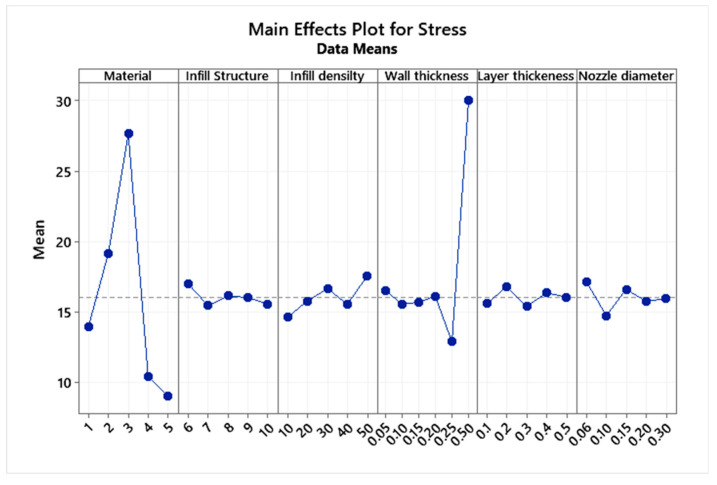
Main effects plot for equivalent stress.

**Figure 8:**
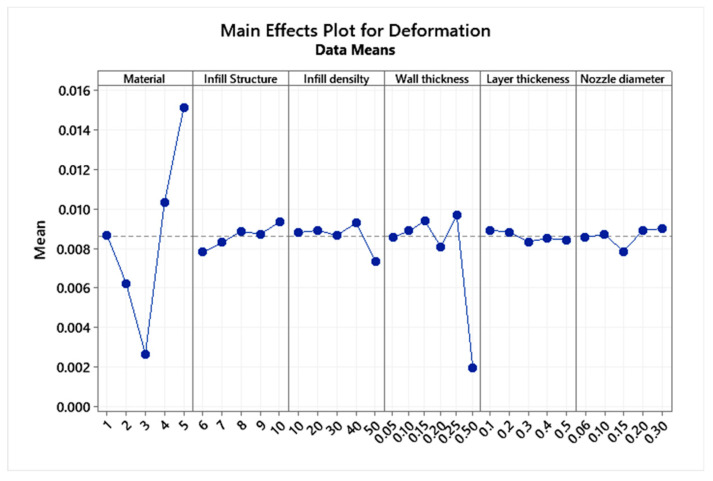
Main effects plot for stress.

Table 5 shows the values of total deformation, maximum stress, and safety factor obtained from the finite element methods and the regression equation, along with the error percentage. The calculated error percentage is below 5%, suggesting the legitimacy of the regression equation. The main effects plot (Figure 6, Figure 7 and Figure 8) was plotted to understand each parameter’s influence on the abovementioned response factors. The graph suggests that the honeycomb structure offers the best aspects of safety and strength, whereas the gyroid provides the best flexibility.

### 4.2. Algorithm Precision by a Confirmatory Test

The results of the training and testing can be seen in Table 4. It can be inferred that Inception offered the highest accuracy and precision with the least training time. Resnet152, on the other hand, provided equally competitive results. However, it took 341 s to train each epoch. Although CNN and MobileNet took less time to train, the results are not promising. One can infer that Inception is the most suitable network architecture for this application. 

**Figure 9:**
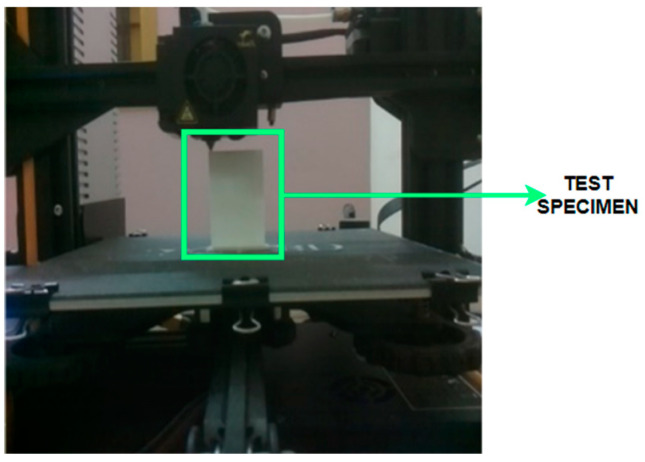
Test specimen.

**Figure 10:**
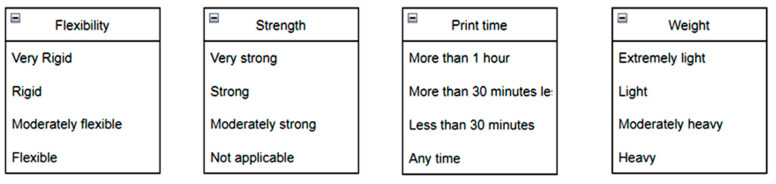
Questionnaire.

**Figure 11:**
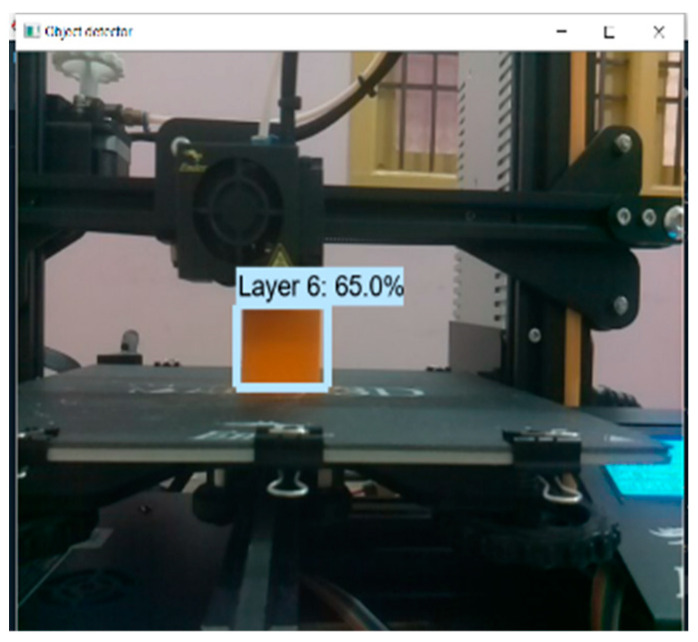
Live testing results.

**Figure 12:**
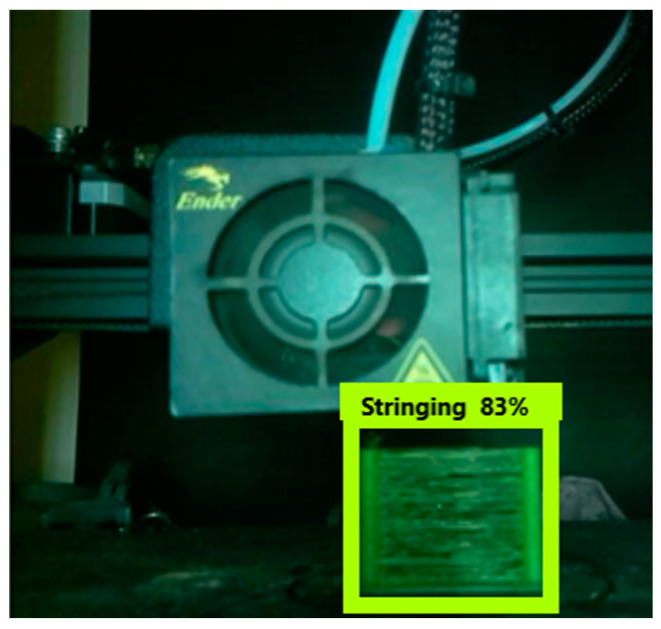
Live testing results with stringing error.

Real-time tests are conducted to find the algorithm’s efficiency and precision. A test specimen shaped like a cuboid (10 × 5 × 2 cm) was chosen to perform the test (Figure 9). Once the application is kicked-started, a standard questionnaire (Figure 10) appears. The user can give their requirements using that questionnaire. The algorithm uses the feed from the user and chooses the appropriate input parameters to be sent to the printer. The tests were performed with and without simulated errors (Figure 11) to test the model’s precision, accuracy, and speed. The time taken to predict must be significantly less, as it can help reduce the wastage of materials. For instance, Figure 12 shows the stringing error. The model identifies the defect and gives counter-commands to rectify the mistake.

## 5. Conclusions

The prediction of optimal input parameters for 3D printing is made by training a machine learning model using the results obtained from finite element analysis. The influence of parameters like material, wall thickness, infill pattern, infill density, nozzle diameter, and layer thickness was investigated using DOE and ANOVA approaches. Based on the former, 25 combinations of those mentioned above were tested, and total deformation, maximum stress, and safety factors were estimated. ANOVA was used to obtain the regression, which was then validated by comparing the values with those obtained from finite element analysis. The regression equations (Equations (1)–(3)) and other important data, including images of simulated errors, were used to train the machine-learning model. The InceptionV3-based algorithm achieved a precision of 100% and an accuracy of 97%. This algorithm helps avoid a similar experiment to predict the optimal input parameters for a given requirement. The algorithm is built so that it requires minimum computational power. Therefore, it can be incorporated into all major manufacturing industries. 

## Figures and Tables

**Table 1:** Thermoplastics’ melting point range.

Thermoplastic	Melting Range (Celsius)
ABS	180–230°
PLA	210–250°
PETG	220–250°
Nylon	240–260°

**Table 2:** Parameter levels.

Symbol	Parameter	Level				
		1	2	3	4	5
X1	Material	1	2	3	4	5
X2	Infill Structure	6	7	8	9	10
X3	Infill density (%)	10	20	30	40	50
X4	Wall thickness (mm)	0.05	0.10	0.15	0.2	0.25
X5	Layer thickness (mm)	0.1	0.2	0.3	0.4	0.5
X6	Nozzle diameter (mm)	0.06	0.1	0.15	0.2	0.3

**Table 3:** DOE table showing different combinations.

Standard Order	Material	Infill Structure	Infill Density	Wall Thickness	Layer Thickness	Nozzle Diameter
1	1	6	10	0.050	0.100	0.060
2	1	7	20	0.100	0.200	0.100
3	1	8	30	0.150	0.300	0.150
4	1	9	40	0.200	0.400	0.200
5	1	10	50	0.250	0.500	0.300
6	2	6	20	0.150	0.400	0.300
7	2	7	30	0.200	0.500	0.060
8	2	8	40	0.250	0.100	0.100
9	2	9	50	0.050	0.200	0.150
10	2	10	10	0.100	0.300	0.200
11	3	6	30	0.500	0.200	0.200
12	3	7	40	0.050	0.300	0.300
13	3	8	50	0.100	0.400	0.060
14	3	9	10	0.150	0.500	0.100
15	3	10	20	0.200	0.100	0.150
16	4	6	40	0.100	0.500	0.150
17	4	7	50	0.150	0.100	0.200
18	4	8	10	0.200	0.200	0.300
19	4	9	20	0.250	0.300	0.060
20	4	10	30	0.050	0.400	0.100
21	5	6	50	0.200	0.300	0.100
22	5	7	10	0.250	0.400	0.150
23	5	8	20	0.050	0.500	0.200
24	5	9	30	0.100	0.100	0.300
25	5	10	40	0.150	0.200	0.060

Where, 1 = ABS; 2 = PLA; 3 = PETG; 4 = Nylon, 5 = Polyvinyl Alcohol Plastic (PVA). 6 = Honeycomb; 7 = Gyroid; 8 = Tri-hexagon; 9 = Grid; 10 = Cubic.

**Table 4:** Hyperparameters of chosen network architectures with results.

Model Parameters	CNN	Resnet152	MobileNet	Inception
Epochs	100	100	100	100
Precision	0.64	0.8	0.63	1.0
Accuracy	0.95	0.95	0.11	0.97
F1 score	0.78	0.64	0.08	1.0
Time taken per epoch	75s	341s	49s	35s
Validation split	0.3	0.2	0.3	0.3
Batch Size	64	32	64	32
Activation Function	BCE	CCE	BCE	SM

**Table 5:** Error analysis.

Standard Order	The Factor of Safety from FEA	Factor of Safety from Equation	Error	Max Equivalent Stress from FEA	Max Equivalent Stress from Equation	Error	Total Deformation from FEA	Total Deformation from Regression Equation (Equations (1)–(3))	Error
1	5.300	5.000	5.600	14.700	15.000	2.000	0.008	0.008	3.600
2	4.500	4.600	2.200	12.100	12.000	0.800	0.009	0.009	3.000
3	5.100	5.000	1.900	14.300	15.000	4.600	0.009	0.009	2.900
4	4.800	5.000	4.100	13.600	14.000	2.800	0.009	0.009	1.700
5	5.700	5.800	1.700	15.200	16.000	5.000	0.008	0.008	1.200
6	6.600	6.900	4.500	19.800	20.000	1.000	0.007	0.007	2.400
7	7.000	7.000	0.000	20.400	20.000	2.000	0.005	0.005	1.700
8	6.400	6.000	6.200	17.300	17.000	1.700	0.007	0.007	1.600
9	8.900	9.000	1.100	22.500	22.000	2.200	0.005	0.005	1.900
10	6.100	6.000	1.600	15.900	15.500	2.500	0.007	0.008	0.500
11	9.700	10.000	3.000	30.000	29.000	3.400	0.002	0.002	2.000
12	9.100	9.000	1.000	26.300	27.000	2.500	0.003	0.003	4.100
13	10.000	10.000	0.000	30.200	30.000	0.600	0.002	0.002	5.500
14	8.900	9.000	1.100	24.600	25.000	1.600	0.004	0.004	2.700
15	9.300	9.000	3.200	27.100	27.000	0.300	0.003	0.003	2.900
16	4.400	4.500	2.200	11.000	11.500	4.300	0.010	0.010	4.100
17	4.100	4.000	2.400	10.300	10.000	3.000	0.010	0.010	1.900
18	3.800	4.000	5.200	9.900	10.000	1.000	0.011	0.011	0.900
19	4.400	4.500	2.200	10.900	11.000	0.900	0.010	0.010	0.300
20	4.000	4.000	0.000	10.100	10.000	1.000	0.011	0.011	0.900
21	3.300	3.400	3.000	9.700	10.000	3.000	0.012	0.013	4.800
22	2.000	2.000	0.000	8.200	8.000	2.500	0.014	0.014	2.100
23	2.400	2.500	4.100	9.100	9.000	1.100	0.016	0.016	1.200
24	2.300	2.400	4.300	8.600	9.000	4.400	0.016	0.016	1.800
25	3.000	3.000	0.000	9.600	10.000	4.000	0.018	0.018	2.800

## Data Availability

Not applicable.

## Nomenclature

CNN	Convolutional Neural Network
RSM	Response Surface Methodology
FFF	Fused Deposition Modelling
PLA	Polylactic acid
ABS	Acrylonitrile Butadiene Styrene
PETG	Polyethylene terephthalate glycol
PVA	Polyvinyl Alcohol Plastic
